# Machine learning approach identifies meconium metabolites as potential biomarkers of neonatal hyperbilirubinemia

**DOI:** 10.1016/j.csbj.2022.03.039

**Published:** 2022-04-02

**Authors:** Shujuan Zeng, Zhangxing Wang, Peng Zhang, Zhaoqing Yin, Xunbin Huang, Xisheng Tang, Lindong Shi, Kaiping Guo, Ting Liu, Mingbang Wang, Huixian Qiu

**Affiliations:** a Division of Neonatology, Longgang District Central Hospital of Shenzhen, Guangdong 518116, China; b Division of Neonatology, Shenzhen Longhua People’s Hospital, Guangdong 518109, China; c Shanghai Key Laboratory of Birth Defects, Division of Neonatology, Children’s Hospital of Fudan University, National Center for Children’s Health, Shanghai 201102, China; d Division of Neonatology, The People's Hospital of Dehong Autonomous Prefecture, Mangshi, Yunnan 678400, China; e Oncology Department, Longgang District Central Hospital of Shenzhen, Shenzhen 518116, China; f Microbiome Therapy Center, South China Hospital, Health Science Center, Shenzhen University, Shenzhen 518116, China

**Keywords:** neonatal hyperbilirubinemia, Gut microbiota, metabolome, Machine learning, causal inference, branched-chain amino acid, BCAA, branched-chain amino acid, NJ, neonatal jaundice, HC, healthy controls, ROC, receiver operating characteristic, AUROC, the area under the ROC, LC-MS, liquid chromatography-mass spectrometry, PCA, the principal component analysis, PLS, partial least-squares regression, OPLS-DA, orthogonal partial least squares-discriminant analysis, MSUD, maple syrup urine disease, KEGG, Kyoto Encyclopedia of Genes and Genomes

## Abstract

**Background:**

The gut microbiota plays an important role in the early stages of human life. Our previous study showed that the abundance of intestinal flora involved in galactose metabolism was altered and correlated with increased serum bilirubin levels in children with jaundice. We conducted the present study to systematically evaluate alterations in the meconium metabolome of neonates with jaundice and search for metabolic markers associated with neonatal jaundice.

**Methods:**

We included 68 neonates with neonatal hyperbilirubinemia, also known as neonatal jaundice (NJ) and 68 matched healthy controls (HC), collected meconium samples from them at birth, and performed metabolomic analysis via liquid chromatography-mass spectrometry.

**Results:**

Gut metabolites enabled clearly distinguishing the neonatal jaundice (NJ) and healthy control (HC) groups. We also identified the compositions of the gut metabolites that differed significantly between the NJ and HC groups; these differentially significant metabolites were enriched in aminyl tRNA biosynthesis; pantothenic acid and coenzyme biosynthesis; and the valine, leucine and isoleucine biosynthesis pathways. Gut branched-chain amino acid (BCAA) levels were positively correlated with serum bilirubin levels, and the area under the receiver operating characteristic curve of the random forest classifier model based on BCAAs, proline, methionine, phenylalanine and total bilirubin reached 96.9%, showing good potential for diagnostic applications. Machine learning-based causal inference analysis revealed the causal effect of BCAAs on serum total bilirubin and NJ.

**Conclusions:**

Altered gut metabolites in neonates with jaundice showed that increased BCAAs and total serum bilirubin were positively correlated. BCAAs proline, methionine, phenylalanine are potential biomarkers of NJ.

## Graphical abstract

**Figure:**
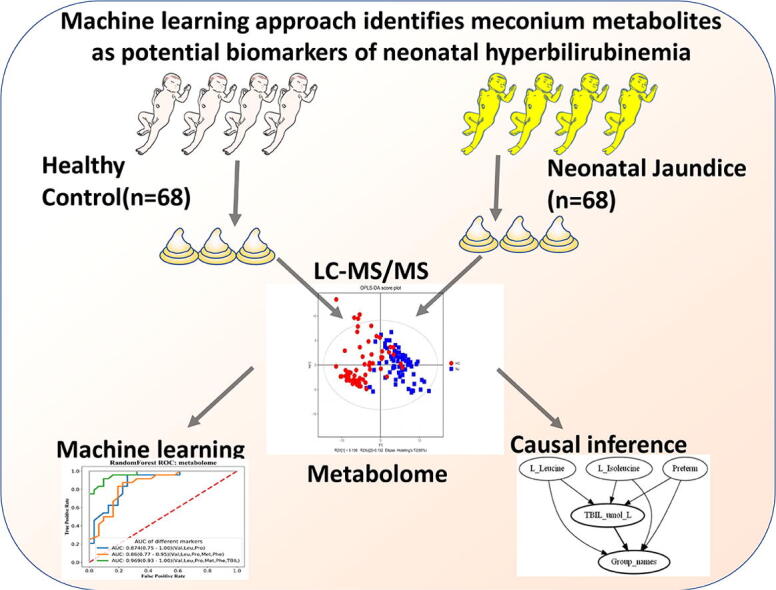

## 1 Introduction

Neonatal hyperbilirubinemia, also known as neonatal jaundice, Jaundice is common in the neonatal period, occurring in 60%–84% of full- and near-full-term neonates, and hyperbilirubinemia occurs in approximately 8%–11% of newborns [1], accounting for 49.1% of hospitalized neonates [2]. Severe hyperbilirubinemia can lead to bilirubin encephalopathy and severe sequelae, imposing a heavy burden on society and families [3], [4].

Studies on neonatal hyperbilirubinemia have shown that the enterohepatic circulation plays an important role in bilirubin excretion [5] and that the gut microbiota is involved in the development of several liver diseases via the gut-liver axis [6], [7]. We previously performed a metagenomic analysis of neonatal jaundice, which showed that changes in the gut microbiotas of patients with jaundice were mainly characterized by significantly decreased abundances of *Bifidobacteria* and galactose-metabolizing bacteria and suggested that *Bifidobacteria* may be involved in bilirubin metabolism via the galactose-metabolizing pathway [8]. Few studies have been published on neonatal metabolomics, and those studies mainly assessed metabolomics from serum samples [9]. Serum metabolite studies in neonates with jaundice have suggested abnormalities in amino acid metabolism in these patients [10] and identified biomarkers that can be used for early diagnosis of biliary atresia, a potential cause of jaundice [9], [11]. Studies on gut metabolomics in neonates with jaundice are lacking.

Previous studies suggested that gut metabolites may play important roles in jaundice and that the gut microbiota is associated with serum bilirubin. In this study, we further investigated the possible mechanisms underlying the development of NJ and bilirubin encephalopathy by examining the differences in gut metabolomics between neonates with and without jaundice.

## 2 Methods

### 2.1 Participants and sample collection

All patients were from a tertiary general hospital in Shenzhen, China. The diagnostic criteria for neonatal hyperbilirubinemia referred to the American Academy of Pediatrics Guidelines for Neonatal Jaundice Intervention [12] and the Expert Consensus on the Diagnosis and Treatment of Neonatal Hyperbilirubinemia of the Neonatology Group of the Chinese Medical Association Pediatrics Branch [13]. Inclusion criteria were no high-risk factors in the mother before birth and no fetal defecation after birth before enrollment. Exclusion criteria were mothers with high-risk factors, antibiotic use within 2 weeks before delivery, newborns who were younger than gestational age, and newborns with severe infections or congenital malformations confirmed after admission. The first meconium samples (3–5 g) excreted by the newborns included in the study after birth were collected in sterile containers by researchers with gloves, avoided inadvertent pollution, then were placed in a − 80℃ freezer immediately. The neonates were grouped into either the NJ or healthy control (HC) group based on their serum bilirubin levels such as TBIL: total bilirubin (UniCel DxC800 Synchron automatic biochemical analysis instrument from Beckman Coulter Co., LTD) during hospitalization. Specimens from patients whose basic data (e.g., sex, gestational age, birth weight and birth mode) did not significantly differ (P > 0.05) were further analyzed. The hospital’s medical ethics committee approved the study protocol, which was performed in accordance with the Declaration of Helsinki. Each child's parents provided written informed consent.

### 2.2 Liquid chromatography-mass spectrometry (LC-MS)

LC-MS was performed as previously described [14], [15]. The collected stool samples were freeze-dried to remove water, then approximately 30 mg of the stool was weighted and added to 600 µL of 50% acetonitrile/water extract containing 5 µM chlorosulfonylurea (internal standard), mixed thoroughly and sonicated at room temperature for 30 min. To better remove impurities from the stool, an equal volume of the extract was added to 200 µL of supernatant from the first centrifugation, vortexed and centrifuged at 18,000 r/min for 25 min. The supernatant was then sampled for analysis. An Ultimate 3000 LC system (Thermo Scientific, Waltham, MA, USA) coupled with an Acquity UPLC HSS T3 column (2.1 mm × 100 mm, 1.8 µm; Waters Corporation, Milford, MA, USA) was used to separate the metabolites. MS was then performed using an Orbitrap Elite mass spectrometer (Thermo Scientific) in electron spray ionization-positive and -negative modes (ESI + and ESI − ) per the manufacturer's instructions.

### 2.3 Metabolomics analysis

Regarding the methodology of metabolomic analysis, we mainly refer to our previous publication [14], [15] and the raw data were preprocessed by Compound Discoverer software (ThermoFisher Scientific, USA) for LC/MS data （detailed in Supplementary Appendix),in short, the extracted data normalized to the sum of the peak area before analysis, multivariate statistical analysis was performed using SIMCA-P Software (Umetrics AB, Umea, Sweden), including PCA analysis, PLS-DA analysis and OPLS-DA analysis. Differential metabolites were screened by OPLS-DA model VIP (variable weight) value > 1 and T-test P value (P < 0.05). After screening the differential compounds, HMDB and KEGG database were used to match the corresponding mass-charge ratio (PPM < 10) to list the candidate compounds. Final matching and identification by the secondary fragment corresponding to the differential compound, Then using KEGG and MetaboAnalyst https://www.metaboanalyst.ca/ https://www.genome.jp/kegg/ commercial database to analyze the metabolite pathways.

### 2.4 Machine learning and causal inference

Machine learning was performed as previously described [14], [16] to determine which meconium metabolites could be used as neonatal hyperbilirubinemia biomarkers. As the random forest method allows for ranking the importance of the selected features.We used the Random Forest Classifier function of scikit learn (version 0.23.1) to determine the importance of the meconium metabolites. We used the train_test_split function (parameter, test_size = 0.4) to split the samples into training and validation sets, then used Random Forest Classifier to train and validate the model for neonatal hyperbilirubinemia classification and used the roc_curve function to plot the receiver operating characteristic (ROC) curve to obtain the area under the ROC (AUROC).

Machine learning-based causal inference was performed using the Microsoft DoWhy (https://github.com/microsoft/dowhy) and EconML (https://github.com/econml/) libraries following the software manual as detailed in another manuscript [16] (detailed in Supplementary Appendix). Briefly, meconium metabolites might lead to NJ was encoded into a causal model and represented by a graph, with each arrow in the graph indicating a causal relationship. Second, Dowhy's backdoor.linear_regression method was used to check whether meconium metabolites could estimate thelevel of TBIL. Third, EconML's machine-learning method was used to construct the estimator using gradient-boosting trees to learn the relationship between the outcome and confounders and the relationship between the intervention and confounders and finally compare the residuals between the outcome and intervention. Finally, placebo_treatment_refuter and data_subset_refuter tests were used to evaluate the model’s robustness.

## 3 Results

### 3.1 Gut metabolomics clearly distinguished neonates with jaundice from HCs

Sixty-eight neonates with hyperbilirubinemia were included in this study: 38 males and 30 females, of whom, 55 were delivered vaginally, and 13 were delivered via cesarean section, mean birth weight of (3144 ± 386) g, mean TBIL of (275.0 ± 64.0) umol/L.Sixty-eight matched HCs were also included: 35 males and 33 females, of whom, 44 were delivered vaginally, and 24 were delivered via cesarean section, mean birth weight of (3096 ± 466) g, mean TBIL of (150.6 ± 40.7) umol/L (Supplementary Table 1). There was no significant difference between the two groups except for serum bilirubin (P < 0.05).

**Fig. 1:**
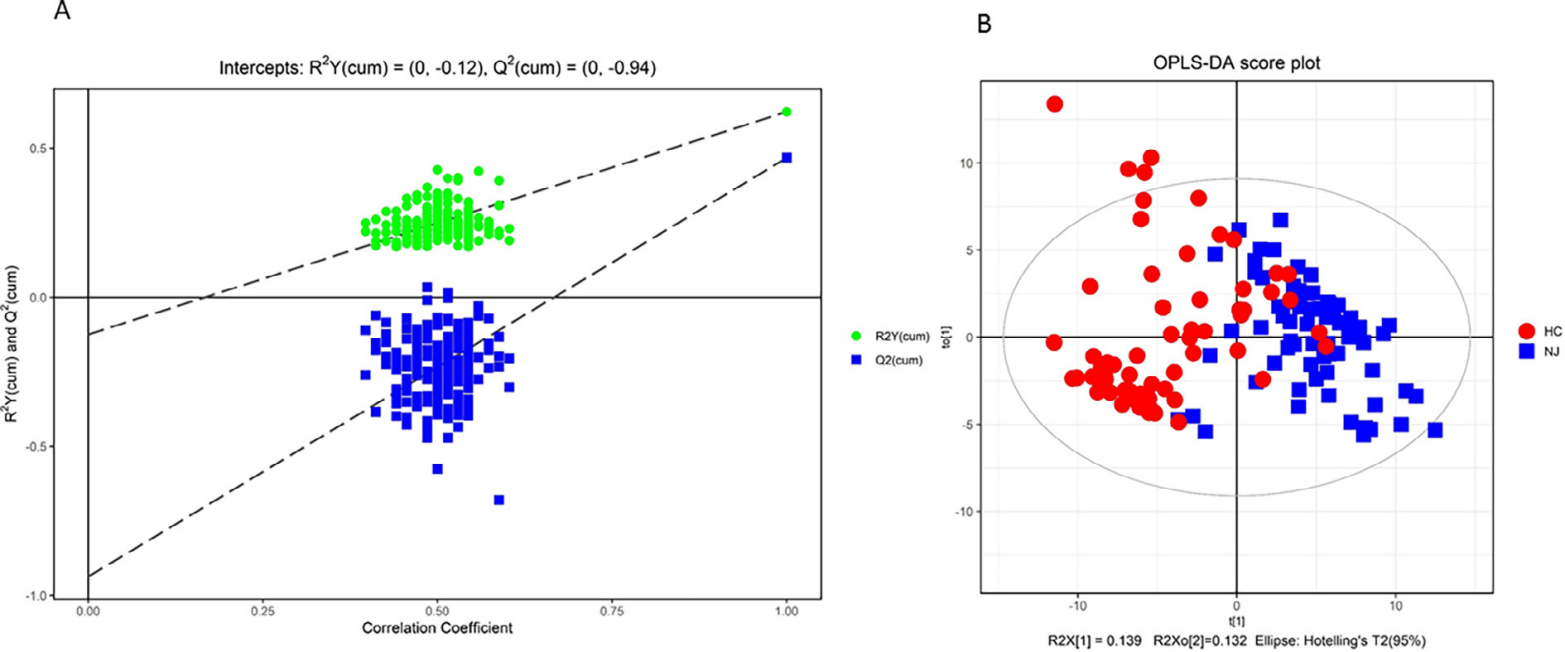
OPLS-DA model to evaluate metabolomic data. A, Permutation test of the OPLS-DA model for the NJ vs HC groups. The original model (R2Y) was closer to 1, indicating that the established model was more consistent with the real situation of the sample data. The original model (Q^2^) was close to 0.5, indicating that adding a new sample to the model yielded a more approximate distribution and that the original model better explained the differences between the two sample groups. **B**, Scatter plot of the OPLS-DA model scores for the NJ vs. HC groups; the two groups of sample metabolites can be clearly distinguished.

The OPLS-DA model was built, and the R^2^ and Q^2^ values were used to test the overfitting of the model and assess its statistical significance. The original model (R2Y) was closer to 1, indicating that the established model was more consistent with the real situation of the sample data. The original model (Q^2^) was close to 0.5, indicating that adding a new sample to the model yielded a more approximate distribution, and the original model better explained the differences between the two sample groups (Fig. 1**A)**. Thus, the original model had good robustness, with no overfitting, and the fecal metabolomic analysis results showed good stability for the samples and instruments in both positive- and negative-ion mode. The metabolites clearly distinguished NJ from HC **(**Fig. 1**B)**.

**Fig. 2:**
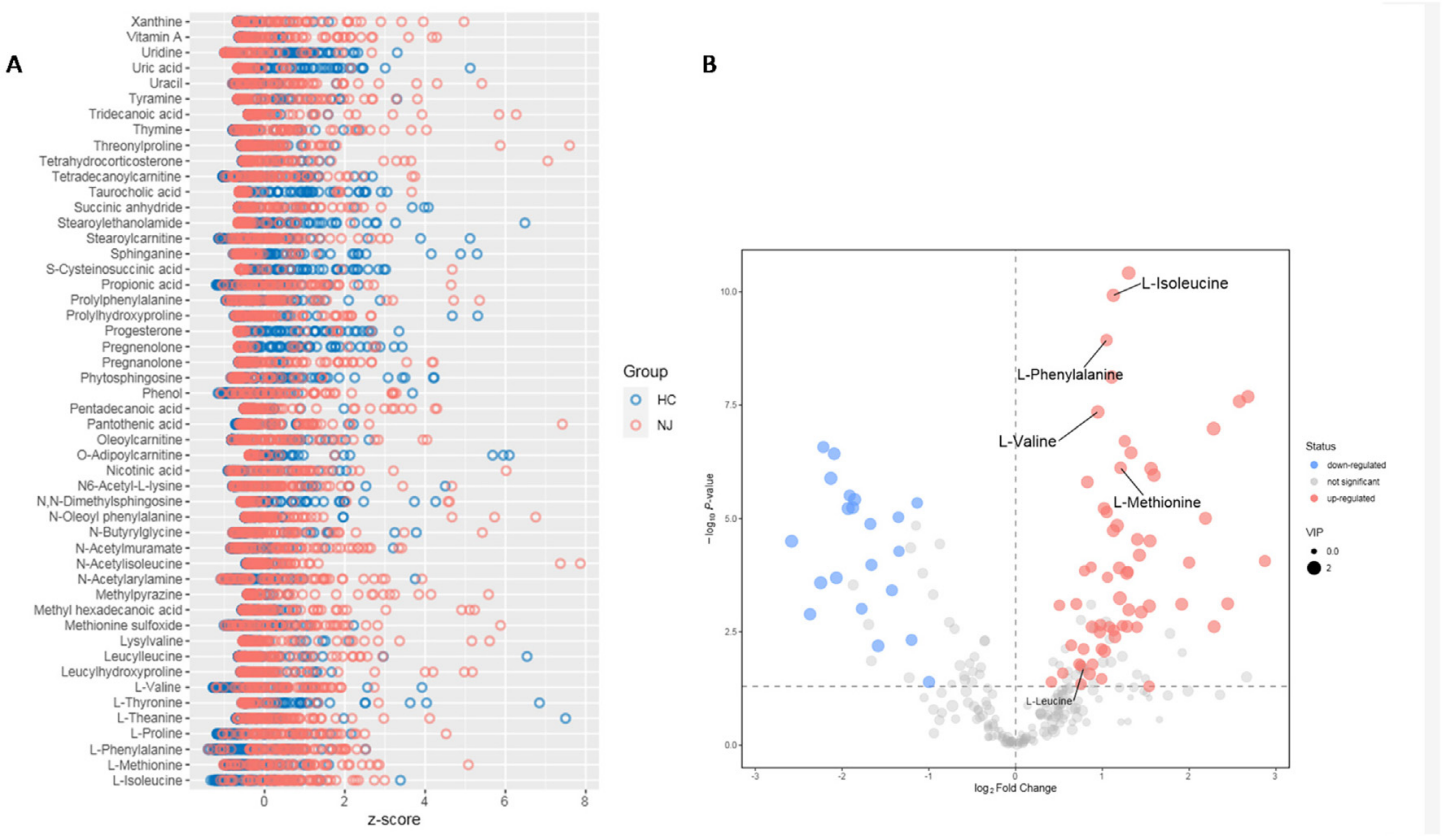
Visualization of metabolites that differed significantly between the NJ and HC groups. A, z-score plots showing the extent of variation in the differentially significant metabolites between the NJ and HC groups. z-score plots show that the metabolites were highly variable across the groups, with z-scores ranging from − 2 to 8 relative to those of the HCs. B, Volcano diagram showing the metabolites that differed significantly between the NJ and HC groups. Each point represents a metabolite; the horizontal coordinate represents the fold change of the group comparing each substance (taken as the logarithm with a base of 2). The vertical coordinate represents the P-value of the Student's *t*-test (taken as the negative logarithm with a base of 10), and the scatter size represents the VIP value of the OPLS-DA model, with a larger scatter indicating a larger VIP value. The scatter color represents the final screening results, with significantly upregulated metabolites in red, significantly downregulated metabolites in blue, and non-significantly different metabolites in gray. (For interpretation of the references to color in this figure legend, the reader is referred to the web version of this article.)

Based on the good robustness of the study model and the likelihood that metabolites can clearly distinguish the NJ group from the HC group, we further visualized the differential metabolites. First, we measured the relative levels of the metabolites at the same level based on z-scores (Fig. 2**A**), and the metabolite groups varied largely across the groups. The z-scores ranged from − 2 to 8 relative to those of the HC group.

We constructed a volcano plot to visualize the metabolites that differed. 82 metabolites differed significantly between the NJ and HC groups, of which, 61 were significantly enriched in the NJ group, including valine, leucine, isoleucine, methionine, and phenylalanine, and 21 were significantly enriched in the HC group. Supplementary Tables 2–4 shows the specific metabolites that differed.

### 3.2 Gut metabolomic characteristics of NJ

**Fig. 3:**
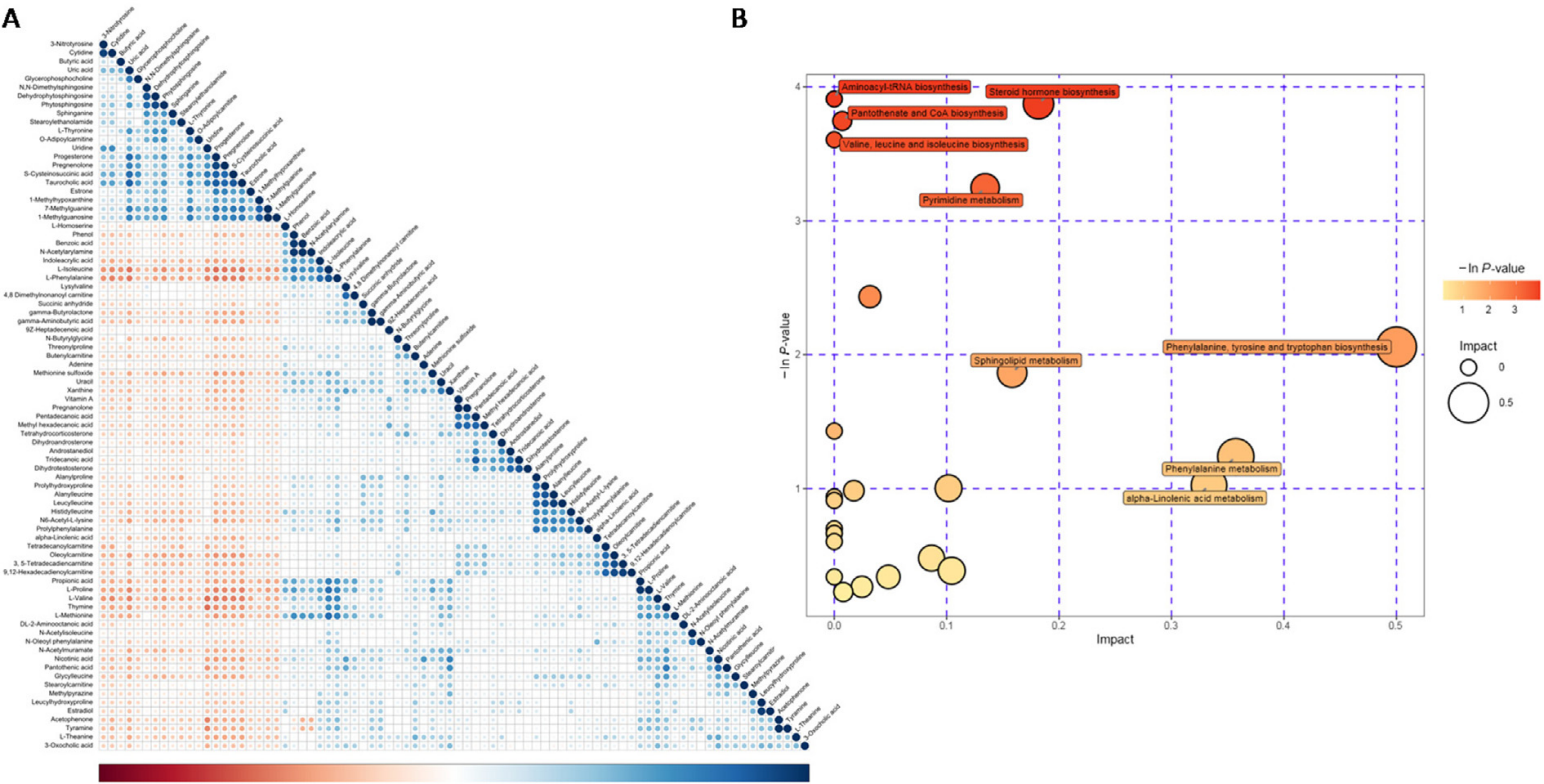
Metabolome characteristics of neonatal jaundice. A, Corrplot of correlations of differential metabolites; corr test P < 0.05 was considered significant. When the linear relationship between two metabolites was enhanced, it tended to be near 1 for a positive correlation and − 1 for a negative correlation. B, Metabolic pathway enrichment bubble plot: the vertical coordinate with the bubble indicates the P-value of the enrichment analysis, taking the negative logarithm of the natural number e as the base (i.e., for the -lnP-value, darker colors indicate a smaller P-value and a more significant enrichment).

To understand the metabolomic characteristics of NJ, we conducted an in-depth analysis of the metabolites that differed between the NJ and HC groups. First, we performed a correlation analysis of the differential metabolites (Fig. 3**A)**. After obtaining the pathway information for the differential metabolites by mapping them against metabolite databases such as KEGG and PubChem, we performed metabolic pathway enrichment analysis using MetaboAnalyst (Fig. 3**B)**. ESI-positive-mode results showed high enrichment of histidyl tRNA biosynthesis; pantothenic acid and coenzyme A biosynthesis; and valine, leucine and isoleucine biosynthesis in the NJ group. ESI-negative-mode pathway enrichment results showed high enrichment of valine, leucine and isoleucine biosynthesis; pyrimidine metabolism; pyruvate metabolism; and valine, leucine and isoleucine degradation (Supplementary Appendix).

### 3.3 Value of gut metabolites for clinical applications

**Fig. 4:**
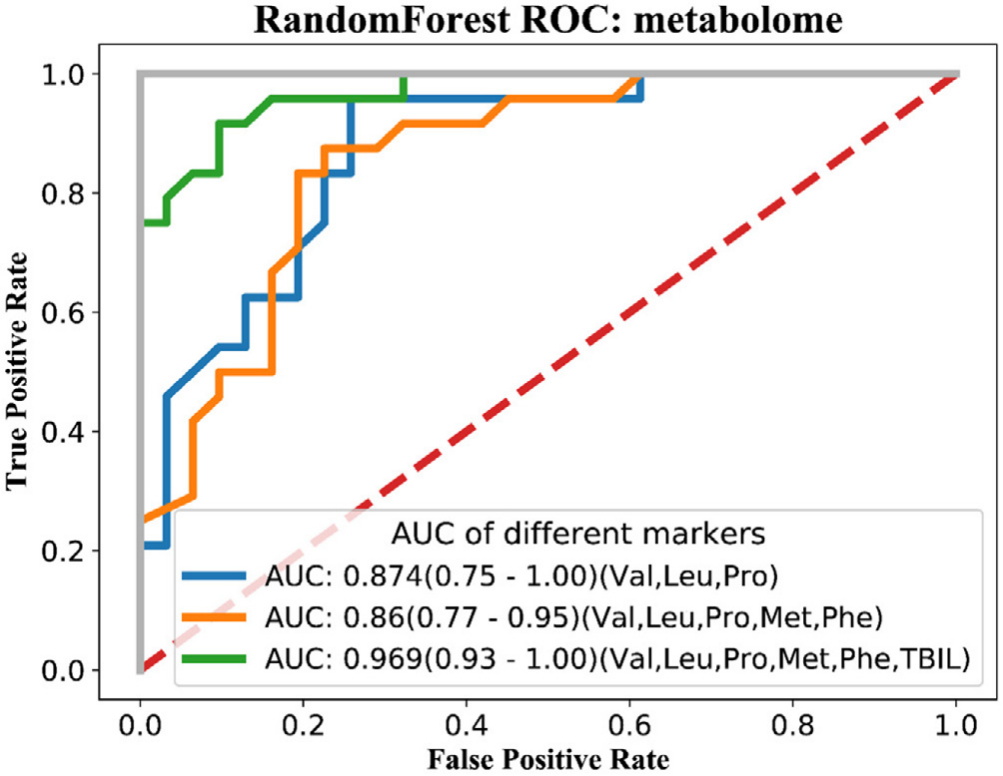
Random forest machine-learning model to assess the value of differential metabolites for clinical applications.

To further assess the value of clinical applications for gut metabolites that differ significantly between neonates with jaundice and HCs, we performed an in-depth analysis based on a random forest machine-learning model. The AUROC score for the differential metabolites (i.e., valine, leucine, and proline) was 0.874; the AUROC score for the combination of methionine and phenylalanine in addition to the above amino acids was 0.86, and the AUROC score for the combination of valine, leucine, proline, methionine, phenylalanine, and bilirubin was 0.969 (Fig. 4). Therefore, the combination of valine, leucine, proline, methionine, phenylalanine and bilirubin represents a potential biomarker for diagnosing NJ.

**Fig. 5:**
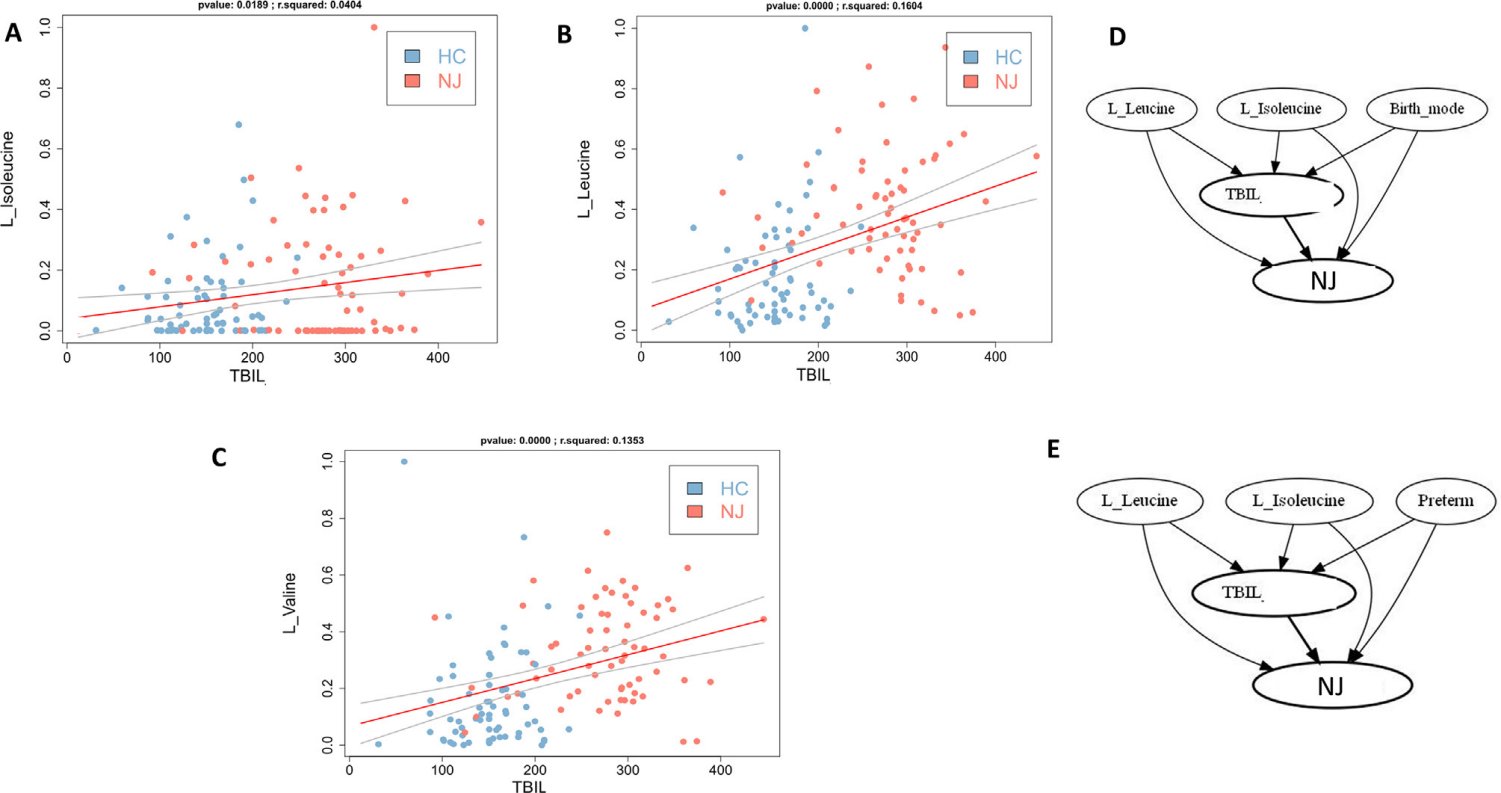
Gut BCAAs have a causal effect on serum bilirubin. A–C, gut branched-chain amino acids isoleucine (A), leucine (B), valine (C) were positively correlated with serum bilirubin levels; D–E, isoleucine, leucine had a direct causal effect on serum bilirubin levels and an indirect causal effect on NJ, birth mode (D) and preterm (E) are confounding factors.

To further understand the biological significance of the metabolomic features of NJ, we evaluated the causal effect of treatment on outcomes based on a machine-learning causal inference approach using differential metabolites as the treatment and important clinical indicators, such as serum bilirubin levels, as outcomes. Notably, the branched-chain amino acids (BCAAs), valine, leucine, and isoleucine, were positively correlated with serum bilirubin (Fig. 5**A–C**), and the BCAAs, leucine and isoleucine, had a direct causal effect on serum bilirubin and thus an indirect causal effect on NJ (Fig. 5**D–E**).

## 4 Discussion

LC-MS is used in metabolomic studies of stool, serum, and other samples to identify metabolites associated with diseases such as genetic metabolic disorders, autism, diabetes, and cancer [17], [18]. Previously, we conducted a study of the gut microbiotas of neonates with jaundice and found that changes in the gut microbiotas in these patients were mainly characterized by significantly decreased abundances of *Bifidobacteria* and gut bacteria involved in galactose metabolism and were positively correlated with increased serum bilirubin levels. To further investigate the roles and mechanisms of the gut microbiota in neonatal hyperbilirubinemia, we conducted the present study to know the gut metabolomics features of neonatal hyperbilirubinemia.

We are aware that classical machine learning methods aim to find feature variables that can be used for disease classification and in this study we used a random forest model mainly because of its ability to rank metabolites. However, once metabolites have been found for disease classification, there is a need to further elaborate on whether there is a potential causal link between metabolites and neonatal hyperbilirubinemia/important clinical features, such as TBIL. The study of causal inference is becoming a current hot topic in artificial intelligence research, and we developed an algorithm for machine learning causal inference, which we found in a preliminary study to identify oral microbes with causal associations with autism from omics data [19]. Based on this, following the identification of potential biomarkers of neonatal hyperbilirubinemia, we performed a machine learning causal inference assessment to further elucidate the molecular mechanisms that are involved in neonatal hyperbilirubinemia occurrence.

We revealed the intestinal metabolomic profile of neonatal hyperbilirubinemia via LC-MS/MS and found that neonates with hyperbilirubinemia and HCs could be clearly distinguished based on their intestinal metabolome via OPLS-DA. For further analysis of differential metabolites, heat map of correlation coefficient of differential metabolites and KEGG annotation were made. Finally, pathway analysis was conducted ESI-positive-mode and negative-mode results both showed high enrichment valine, leucine and isoleucine biosynthesis in the NJ group. Notably, NJ group manifested as a significant elevation of intestinal BCAAs (include leucine, isoleucine and valine, which are nutritionally essential amino acids), proline, methionine, phenylalanine and were positively correlated with elevated total serum bilirubin.

Previous studies have shown that elevated valine, leucine, lysine, isoleucine and alanine levels reflected abnormal amino acid metabolism in patients with neonatal jaundice [10], and revealed transient high serum methionine levels and hypermethioninemia helped distinguish the various causes of obstructive jaundice in these infants [20]. Additionally, altered serum phenylalanine, ornithine, isoleucine and leucine metabolism in neonates is closely associated with the presence of combined bilirubin encephalopathy in neonates with hyperbilirubinemia [21], which is consistent with the results of our study. Whether the results of our gut metabolomic may help distinguish neonates with hyperbilirubinemia from HC and as metabolic markers for NJ requires further investigation.

BCAAs are involved in the synthesis of cholesterol, ketone bodies and glucose as the basic units of synthetic peptide chains [22], [23], [24], [25]; and also affect protein metabolism, especially leucine and its metabolites [26]. Increased BCAA levels in a group of pregnant women with gestational diabetes mellitus were positively corretlated with glucose metabolism and lipid metabolism disorders [27], [28], [29], which could be used to objectively reflect the severity of gestional diabetes or as early biomarkers of disease onset [30]. Our study showed abnormalities in BCAA metabolism in NJ group, suggesting that BCAAs in the meconium could also be a potential risk factor for neonatal hyperbilirubinemia.

So we performed an indepth analysis based on a random forest machine-learning model. Our study showed an AUROC score of 0.874 for the differential metabolites, valine, leucine, proline; and AUROC score of 0.969 for the combination of valine, leucine, proline, methionine, phenylalanine and bilirubin was reliable in diagnosing neonatal hyperbilirubinemia. Hence, the combination of valine, leucine, proline, methionine, phenylalanine and bilirubin was reliable in diagnosing neonatal hyperbilirubinemia. Based on machine learning, BACCs may play important roles in neonatal hyperbilirubinemia and the ROC value may predict the diagnosis.

Here, we collected the first postnatal stool, which reflected the prenatal and immediate postnatal metabolic status. Starvation [31], [32] and inadequate intake [33], [34], [35] or high blood glucose can lead to increased BCAAs [36], [37], [38], suggesting that the increase in fecal BCAAs may be due to maternal starvation, inadequate intake or high blood glucose. Further studies on maternal illness and prenatal intake status are needed.

Since serum BCAA abnormalities are potential disease biomarkers. Previous studies show that high BCAA levels are associated with excitotoxicity, energy deficiency and oxidative stress in the brain [39], [40], leading to severe neurological symptoms [41], [42], [43]. DNA damage in the hippocampus and striatum was confirmed after administering BCAAs in an animal model of maple syrup urine disease (MSUD) [29], [44]. These studies suggest that increased protein breakdown or decreased protein synthesis [26], [45], [46] in the muscles and insulin resistance may enhance BCAA levels. However, levels that are too high can damage the nervous system. This may explain the predisposition to brain damage in neonates with severe hyperbilirubinemia, leading to the development of bilirubin encephalopathy. This requires further research.

And our previous study found that patients with NJ exhibited a reduction in bifidobacteria [8], which are involved in amino acid metabolism [47], although there is little evidence for the involvement of bifidobacteria in branched-chain amino acids. Bifidobacteria are part of the core microbiota of the healthy infant gut and may form biofilms on intestinal epithelial cells, mucosa and food particles [47]. Faizan et al. found that Bifidobacterium may be involved in the metabolism of branched-chain amino acids, and they found that relative to other non-bifidobacteria, bifidobacterial biofilms involved in amino acid metabolism, particularly branched-chain amino acid gene [48]. However, we need to exclude other effects on branched-chain amino acid metabolism such as starvation or blood glucose and further elaborate on the potential mechanistic link between Bifidobacterium and BCAA production/degradation capacity, which is our next step to be undertaken.

This study identified an alteration in branched-chain amino acid metabolism in neonatal jaundice and independent validation is needed to determine whether this alteration is a biomarker for neonatal jaundice. We will recollect samples and perform targeted metabolomic analysis in future.

## 5 Conclusion

Our results showed that the metabolome clearly distinguished the NJ group from the HCs, with a model AUROC score of 96.9 %. NJ manifests as an increase in intestinal BCAAs and was positively correlated with serum bilirubin. BCAAs have a potential causal effect on serum bilirubin and BCAAs (i.e., valine, leucine and isoleucine) proline, methionine, phenylalanine are potential markers of neonatal hyperbilirubinemia.

## 6 Ethics approval and consent to participate

The study was approved by the Ethics Committee of Longgang District Central Hospital of Shenzhen (NO.2019ECYJ026).

## Authors contributions

All authors designed and executed the study and wrote the manuscript. All authors read and approved the final manuscript.

Under supervision by Huixian Qiu and Mingbang Wang, Shujuan Zeng , Zhangxing Wang , Peng Zhang, Zhaoqing Yin performed data analysis manuscript drafting. Xunbin Huang,Xisheng Tang, Lindong Shi, Kaiping Guo, Ting Liu performed sample preparation and clinical evaluation.

## Declaration of Competing Interest

The authors declare that they have no known competing financial interests or personal relationships that could have appeared to influence the work reported in this paper.
