# Supplementary material for: Machine learning approach identifies meconium metabolites as potential biomarkers of neonatal hyperbilirubinemia
Source: Comput Struct Biotechnol J. 2022 Apr 2;20:1778–84. doi: 10.1016/j.csbj.2022.03.039 (PMC9027383; doi:10.1016/j.csbj.2022.03.039)
Supplement: Supplementary data 2 [file mmc2.docx]

**Supplementary Appendix**

**1 random classifier models metrics for accuracy, precision, recall and F1 scores**

1. Val, Leu, Pro

precision recall f1-score support

0 0.81 0.78 0.79 32

1 0.71 0.74 0.72 23

accuracy 0.76 55

macro avg 0.76 0.76 0.76 55

weighted avg 0.77 0.76 0.76 55

1. Val, Leu, Pro, Met, Phe

precision recall f1-score support

0 0.81 0.81 0.81 31

1 0.75 0.75 0.75 24

accuracy 0.78 55

macro avg 0.78 0.78 0.78 55

weighted avg 0.78 0.78 0.78 55

1. Val, Leu, Pro, Met, Phe, TBIL

precision recall f1-score support

0 0.90 0.90 0.90 31

1 0.88 0.88 0.88 24

accuracy 0.89 55

macro avg 0.89 0.89 0.89 55

weighted avg 0.89 0.89 0.89 55

**2 Internal standard EIC**

Positive mode_ A02 sample internal standard extraction chromatogram

Negative mode_ A02 sample internal standard extraction chromatogram

**3 Metabolomics analysis**

First of all, the raw data were preprocessed by Compound Discoverer software (ThermoFisher Scientific, USA) for LC/MS data where parameters were as follows:intensity threshold,30,000m/z range,50-1000m/z width,5 ppm;frame time width,0.2 min;retention time start and end values were 0.01-19.5 min. A total of 3725 features were extracted，the extracted data was normalized to the sum of the peak area before analysis .Then imported the processed data in SIMCA-P software for principal component analysis (PCA) and orthogonal projections to latent structures-discriminate analysis (OPLS-DA). Principal component analysis (PCA) showed the distribution of origin data. In order to obtain a higher level of group separation and get a better understanding of variables responsible for classification, supervised orthogonal projections to latent structures-discriminate analysis (OPLS-DA) were applied. Afterwards, the parameters for the classification from the software were R2Y = 0.622 and Q2Y = 0.468, which were stable and good to fitness and Statistical. 7-fold cross validation was used to estimate the robustness and the predictive ability of our model, such permutation test was proceeded in order to further validate the model. The R2 and Q2 intercept values were -0.12 and -0.89 after 200 permutations. The low values of Q2 intercept indicate the robustness of the models, and thus show a low risk of over fitting and reliable. Based on the orthogonal projections to latent structures-discriminate analysis (OPLS-DA), a loading plot was constructed, which showed the contribution of variables to difference between two groups. It also showed the important variables which were situated far from the origin, but the loading plot is complex because of many variables. To refine this analysis, the first principal component of variable importance in the projection (VIP) was obtained. The VIP values exceeding 1 were first selected as changed metabolites.In step 2, the remaining variables were then assessed by Student’s t-test (P-value < 0.05), variables were discarded between two comparison groups. In addition, commercial databases including KEGG http://www.genome.jp/kegg/ and MetaboAnalyst http://www.metaboanalyst.ca/ was utilized to search for the pathways of metabolites.

4 Machine learning and causal inference

Causal inference based on machine learning by Microsoft company DoWhy library (https://github.com/microsoft/dowhy) and EconML library (https://github.com/econml/), and refer to the software specification. In the first step( Modeling), we coded our domain knowledge into A causal model, represented by A graph. Each arrow in the graph represents the causal relationship: "A->B" indicates that variable A causes variable B.the outcome is set as a binary variable of group name, where 0 represents the control group and 1 represents the disease group. The variable of treatment is set as an important clinical indicator potentially leading to disease. Other variables to be examined may be common cause variables of treatment and outcome as Confounders. The second step(Identification) is the Backdoor.Linear_regression method based on Dowhy, which checks whether a given observational variable can estimate the target quantity. The third step(Estimation) is to build an Estimator to calculate the Estimand identified identified in the previous step. In order to model nonlinear data (as well as data with high-dimensional confounding factors), we use EconML's machine learning method to build estimator, which uses gradient Boosting trees to learn the relationship between results and confounding factors, as well as the relationship between intervention and confounding factors. Finally, the residuals between the results and the intervention were compared. The fourth step(Refutation)is to check the robustness of the estimates. This is the most important step in causal inference analysis and can be relied on refutation tests, which use an Estimator attribute to refute the correctness of the obtained estimates, given the absence of a suitable validation set. Here, we performed placebo_treatment_refuter, which checks whether the estimator returns an estimate close to zero when the intervention variable is replaced by a random variable, and the subdata_subset_refuter, That is, whether the estimated value obtained by random sampling of some samples (subset_fraction=0.8) is close to the estimated value of all samples.

**5 Supplementary figure**

**
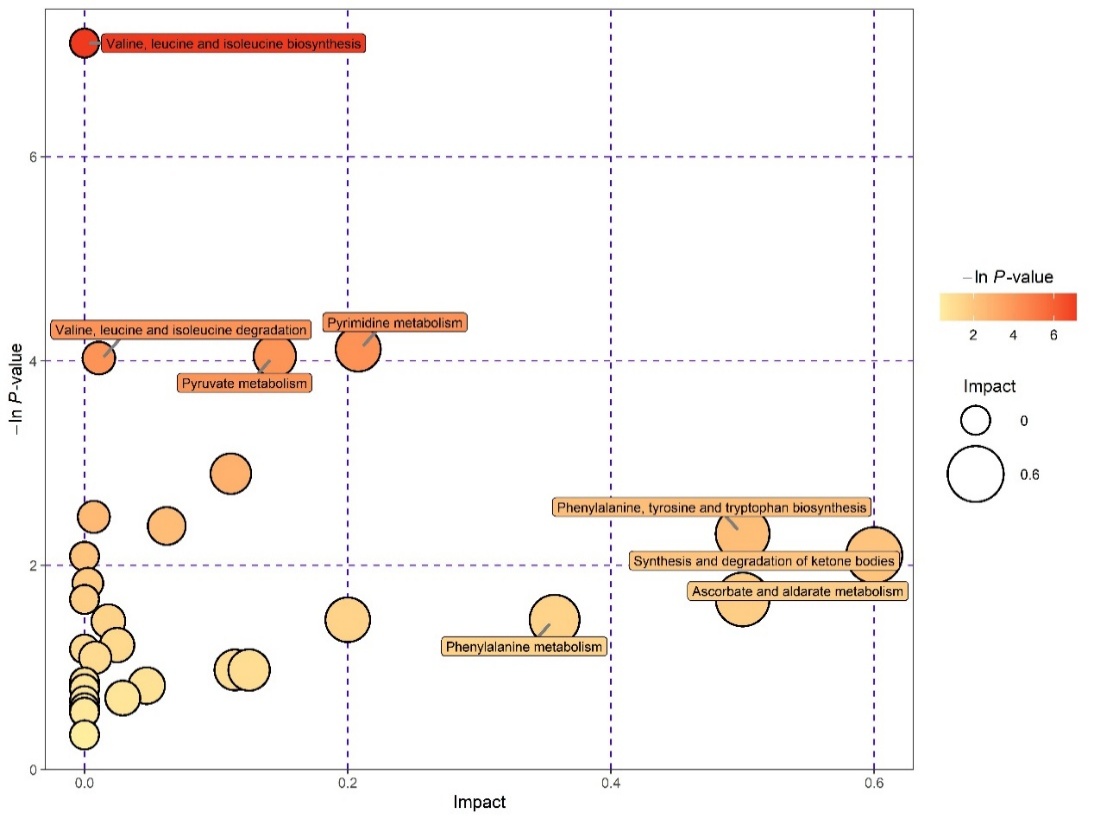
**

**Supplementary Fig1 NEG-Bubble Plot ESI-negative-mode pathway enrichment results showed high enrichment of valine, leucine and isoleucine biosynthesis; pyrimidine metabolism; pyruvate metabolism; and valine, leucine and isoleucine degradation**

**Supplementary Fig2 PCA score plot with quality control samples**
